# Body Coloration Characterization and Proteomic Analysis of Diurnal Color Variation in Farmed *Larimichthys crocea*

**DOI:** 10.3390/ani16030353

**Published:** 2026-01-23

**Authors:** Na Lin, Junjie Wu, Hongjin Deng, Jinli Wang, Banghong Wei, Yao Zheng, Quanyou Guo

**Affiliations:** 1 East China Sea Fisheries Research Institute, Chinese Academy of Fishery Sciences, Shanghai 200090, China; lina903368043@163.com (N.L.); wujj@ecsf.ac.cn (J.W.); denghongjin1020@163.com (H.D.);; 2 College of Ocean Studies, Ningde Normal University, Ningde 352100, China

**Keywords:** *Larimichthys crocea*, body coloration, chromatophores, carotenoids, diurnal body color variation

## Abstract

The large yellow croaker (*Larimichthys crocea*) is an economically significant aquaculture species in Asia, with skin coloration being a key quality attribute affecting consumer choice and commercial value. This study integrated morphological, biochemical, and proteomic approaches to investigate chromatophore characteristics, pigment composition, and diurnal body color variation in the large yellow croaker. Colorimetric analysis showed the highest yellowness, redness, and lightness values in ventral skin, followed by caudal skin, with dorsal skin showing the least yellowness. Three major chromatophores were identified: melanophores, xanthophores, and iridophores. Xanthophores were abundant in ventral and caudal skin, showing larger size and deeper pigmentation ventrally, while melanophores dominated dorsally and iridophores were mainly on scales. Skin and fins were key sites for carotenoid deposition, with lutein as the predominant carotenoid. Esters of lutein and zeaxanthin were the main contributors to yellow coloration in fish. A strong positive correlation was found between skin yellowness and total carotenoid content (*R* = 0.91, *p* < 0.05). The aggregation and dispersion of xanthosomes within xanthophores appeared to be the key driver behind the light-influenced diurnal body color variation. Proteomic analysis revealed motor proteins (such as KIF21, DYNC1L1, and MYO9) and pathways related to intracellular granule movement were probably related to the pigment transport in chromatophores of large yellow croaker. In addition, tubulin dynamics also appeared to participate in pigment translocation. This study is helpful to understand the pigmentary basis of this important farmed sea fish and provides a foundation for improving the golden color traits and further molecular research on diurnal color changes in large yellow croaker.

## Simple Summary

The golden color of the farmed large yellow croaker is highly prized by consumers, directly affecting its market price. This study investigated what gives this fish its yellow coloration and why its color can change between day and night. We found that special yellow pigment cells (xanthophores), containing pigment granules called carotenoids (mainly lutein), are most abundant in its ventral and caudal skin. The fish’s diurnal color change is driven by the movement of these yellow pigments granules (xanthosomes) within the cells, likely controlled by specific motor proteins. This research helps us understand the biology behind the fish’s coloration. The findings provide a scientific basis for aquaculture breeders to improve the desirable golden color in this important seafood species through selective breeding or feed, ultimately delivering a better product to the market.

## 1. Introduction

The large yellow croaker (*Larimichthys crocea*) is one of the four major marine products in China, belonging to the family Sciaenidae within the order Perciformes. It is renowned for its delicious meat, rich nutritional value, and golden yellow body color, symbolizing “wealth”, and holds high economic value. Currently, its aquaculture in China has developed into a large-scale industry. The farming models are transitioning from inshore to offshore deep-water cages to enhance quality and yield. Its national aquaculture production reached 292,600 tons in 2024 [1]. The yellowness of its skin has become one of the important indicators for its quality grading [2]. The dorsal skin of the cultured large yellow croaker is light black, and the skin on the abdomen and caudal fin is orange-yellow. The manifestation of body coloration is closely associated with the type, quantity, shape, and distribution of chromatophores, as well as the distribution and content of pigment [3,4]. Chromatophores can be grouped into two primary types based on optical properties: light-absorbing pigment cells (including melanophores, xanthophores, erythrophores, and cyanophores) and light-reflecting pigment cells (leucophores and iridophores) [5]. Melanophores and xanthophores are widely distributed in teleost fishes [6], such as Zebrafish (*Danio rerio*) [7] and turbot (*Scophthalmus maximus*) [8]. The skin of crucian carp (*Carassius auratus*) also contains erythrophores, a type of red pigment cell [9]. The chromatophores in large yellow croaker mainly contains melanophores (black-brown), xanthophores (yellow), and iridophores [10,11]. The golden-yellow skin coloration in large yellow croaker principally arises from the dense accumulation of carotenoids (pigment granules) within dermal xanthophores [12]. Pigment quantification in the skin of large yellow croaker revealed that the lutein accounted for 50% of total pigments, with minor amounts of canthaxanthin and astaxanthin also detected [13]. Although current studies about chromatophores and pigments in large yellow croaker exist, the distribution patterns of chromatophores, pigment composition, and their relationships with body coloration remain poorly documented.

The regulation of fish body coloration is influenced by multiple factors, with underlying mechanisms categorized into morphological changes and physiological changes. Morphological changes primarily refer to variations in pigment content and chromatophore dynamics, encompassing both the absolute quantities and relative ratios of different pigments, as well as the migration, metabolism, differentiation, proliferation, and apoptosis processes of pigment cells in the integument [14]. These alterations constitute a gradual biological progression. Physiological color changes primarily result from the dispersion and aggregation of pigment granules within chromatophores or the movement of intracellular reflective structures. These processes are regulated by the nervous and endocrine systems and occur rapidly, enabling instantaneous color shifts [15]. Factors influencing the body color of fish include genetics, environment [10,16,17], and aquaculture conditions [18,19,20], among other factors. In the natural environment, coloration is intrinsically linked to fitness and survival. Key environmental parameters such as light spectrum, background color, and water quality can profoundly alter pigmentation. For instance, fish can physiologically adapt their skin darkness and pattern to match their background for camouflage [21], and specific light wavelengths have been shown to enhance pigmentation and improve physiological condition in species like tilapia [22]. Pigment content-induced changes in fish body coloration are the most extensively studied morphological alterations. Carotenoids are a group of natural pigments primarily obtained from the diet, responsible for the yellow, orange, and red coloration in fish [23]. In aquaculture, the deposition of carotenoids in skin and muscle is a crucial economic trait, directly linked to market value and consumer preference [23], since many fish cannot synthesize certain carotenoid and instead metabolize dietary pigments into specific forms [24]. Therefore, in aquaculture, synthetic pigments or natural pigment sources are commonly added to feed to meet fish pigment requirements and enhance body coloration [23,25]. For farmed large yellow croaker, whose distinctive golden hue is a key quality marker, dietary carotenoid supplementation has been identified as an effective method to improve skin pigmentation. Dietary supplementation of astaxanthin in large yellow croaker feed enhanced ventral yellowness, while lutein addition demonstrated a more pronounced effect on increasing ventral yellowness at equivalent dosage levels compared to astaxanthin [20,26]. The addition of pigments to feed primarily improves fish body coloration by increasing the pigment content within chromatophores or directly depositing in tissues. However, the precise composition and concentration of pigments contributing to the distinctive coloration in large yellow croaker remain insufficiently characterized.

The mechanism of light-regulated body color changes in large yellow croaker relies on the centripetal contraction and centrifugal dispersion of pigment granules within chromatophores. Researches reveal this process is dynamically regulated by the microtubule system [27,28]. Post-translational modifications of tubulin precisely control pigment transport by modulating microtubule stability and their interactions with motor proteins [29]. Specifically, dynein drives pigment granule aggregation toward the cell center through ATP hydrolysis [29,30], while kinesin mediates their peripheral dispersion [31,32]. The microfilament system serves as an auxiliary transport pathway that maintains basic translocation function even during microtubule depolymerization [33]. These two systems work synergistically to rapidly adjust pigment distribution, enabling efficient environmental adaptation of body coloration. Under dark conditions, partial exposure of large yellow croaker to white light induces a color shift from yellowish to silvery white in illuminated areas. Under white-light conditions, partial shading of the body surface triggers a reversal from silvery white to yellow in shaded regions [34]. The yellow coloration of large yellow croaker skin is primarily due to the dispersion of xanthosomes within xanthophores, which is regulated by melanocyte-stimulating hormone (MSH) [10]. Meanwhile, the light-induced aggregation of xanthosomes was mediated through Ca^2+^-PKC and Ca^2+^-CaMKII signaling pathways and was dependent on microtubules and dynein [34]. Although the cytoskeletal mechanisms and signaling pathways underlying rapid color change have been elucidated, the proteomic profile and key regulators underlying diurnal body color variation remain unknown, prompting our use of quantitative proteomics to characterize global protein expression dynamics in the large yellow croaker.

Body color serves as a crucial indicator of health status and product quality in aquatic species, directly reflecting the freshness and market value [35], and strongly influencing consumer purchasing behavior. The golden-yellow coloration on the body of large yellow croaker is influenced by light exposure. To preserve this trait, harvesting is typically conducted during nighttime operations. However, such practices involve considerable safety risks for fishermen.

Thus, this study aims to: (1) characterize the association between body coloration and the distribution of chromatophores and pigment content in the large yellow croaker; (2) compare proteomic profiles of ventral skin and scales between day and night conditions, identify key proteins regulating diurnal color change, and explore the potential molecular mechanisms driving this rhythm. The findings will provide a theoretical foundation for improving golden-yellow pigmentation in cultured large yellow croaker and optimizing aquaculture strategies to enhance product quality.

## 2. Materials and Methods

### 2.1. Experimental Fish

The large yellow croaker was cultured at aquaculture farms (multi-connected cage culture mode) in the Ningde, Fujian province, China. Fish (*n* = 26; body weight: 575.06 ± 47.19 g; body length: 31.02 ± 1.56 cm) were caught during the day (14:00) and at night (19:30), respectively. Large yellow croakers designated for chromatophore observation, color difference measurement, and pigment quantification were euthanized by iced-water anesthesia. Fish were kept in a dark place layered with ice, then transported to the laboratory for subsequent analyses. Fish used for proteomic analysis were divided into four groups (*n* = 3 per group): the ventral skin tissues of fish caught at night (YSK) or caught in the daytime (SSK); the scale tissues of fish caught at night (YSC) or caught in the daytime (SSC). After the fish were anesthetized with 3-aminobenzoic acid ethyl ester methanesulfonate (MS-222, 100 mg/L, Sigma, St. Louis, MO, USA), the dermal mucus was wiped off, then scales and skin tissues were collected into centrifuge tubes, flash-frozen in liquid nitrogen, and stored at −80 °C for subsequent analysis.

### 2.2. Color Difference Measurement

**Figure 1:**
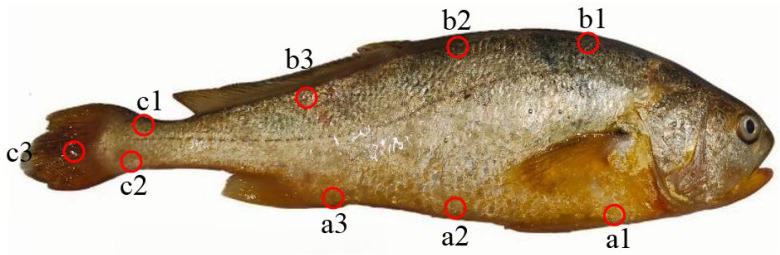
Schematic diagram of body color measurement sites in the large yellow croaker. Ventral area: a1, a2, a3; dorsal area: b1, b2, b3; caudal peduncle: c1, c2, c3. Scale bar = 1:2.71.

Upon arrival at the laboratory, ten large yellow croakers (fish caught at night) were randomly selected. After surface moisture and mucus were removed with swabs, colorimetric values were measured at designated body regions using a chromameter (CR400, Konica Minolta, Tokyo, Japan): ventral area (a1–a3; a1 and a3 refer to the front end of the ventral fin and the anal fin, respectively; a2 refers to the area between a1 and a3), dorsal area (b1–b3; b1–b3 refers to the sites of the dorsal area corresponding to the ventral area), and caudal peduncle (c1–c3) [2], as illustrated in Figure 1. The colorimetric value for the ventral area was represented by the average of sites a1, a2, and a3. Similarly, the dorsal and caudal areas were represented by the averages of sites b1–b3 and c1–c3, respectively. Parameters of L* (lightness), a* (red–green axis), and b* (yellow–blue axis) were measured based on the CIE 1976 standard [36].

### 2.3. Microscopic Observation of Chromatophores

Six large yellow croakers were selected for chromatophores observation. Scale sampling followed this protocol: after surface moisture and mucus were removed with swabs, 4~5 scales were extracted from each designated region (ventral a1–a3, dorsal b1–b3, and caudal c2, Figure 1) using sterile forceps. The scales were rinsed in phosphate-buffered saline (1 × PBS) buffer, mounted on glass slides, and examined under a microscope with photographic documentation. Skin samples (1 cm × 1 cm × 1.5 mm) were excised from the ventral, dorsal, and caudal regions using scissors and forceps, while fin clips (1 cm^2^) were collected with scissors. All samples were examined under a BX53 biological microscope (Olympus Corporation, Hachioji, Japan) for chromatophores analysis.

When observing chromatophores in the ventral skin of the large yellow croaker, pigment cells were counted according to the five-point sampling method [11]. The average area of a single xanthophore was determined by calculating the mean area of five xanthophores per image using ImageJ software (Version number: ImageJ 1.50i). The density of xanthophores was derived by counting the number of cells per unit area, also measured with ImageJ.

### 2.4. Determination of Total Carotenoid Content

Four large yellow croakers were sampled for total carotenoid content quantification, following the method of Yi et al. [26] with minor modifications. Briefly, approximately 0.20 g of each tissue sample (ventral skin-a2, dorsal skin-b2, and caudal skin-c2 in Figure 1; dorsal muscle, ventral muscle, intestine, liver, spleen, and eye) was weighed in centrifuge tubes for total carotenoid quantification. Samples were homogenized in 8 mL ethyl acetate:absolute ethanol (1:1, *v*/*v*) for 1 min using a tissue homogenizer, followed by centrifugation at 4000× *g* for 5 min. The supernatant was collected, and the pellet was sequentially extracted with 4 mL ethyl acetate and 8 mL *n*-hexane. Combined supernatants were evaporated under high-purity nitrogen. The residue was redissolved in 4 mL of acetone containing 0.05% butylated hydroxytoluene (BHT), vortexed, and centrifuged at 10,000× *g* for 5 min. Full-wavelength scanning was performed to identify the characteristic absorption peak, followed by absorbance measurement at the determined wavelength. The total carotenoid concentration was calculated according to the following equation:

$$C=\frac{10,000\times V\times A}{W\times E}$$

where *C* is the total carotenoid concentration (mg/kg wet weight); *V* is the total volume of the extract (mL); *A* is the absorbance value of the solution; *W* is the weight of tissue sample (g); and *E* is the extinction coefficients, with a value of 2500.

### 2.5. Composition Analysis of Carotenoids by UPLC-MS/MS

Ventral skin samples (a2, Figure 1) from large yellow croakers (*n* = 3) were collected for carotenoid composition and content analysis, following the ultra-performance liquid chromatography-tandem mass spectrometry (UPLC-MS/MS) method established by Rodrigues et al. [37] and Zheng et al. [38]. Skin samples were pulverized using a tissue homogenizer (FJ-200, Shanghai HUXI Industrial Co., Ltd., Shanghai, China), and 50 mg of the powdered sample was extracted with 0.5 mL of *n*-hexane/acetone/ethanol (1:1:1, *v*/*v*/*v*) containing 0.01% BHT. After vortex mixing for 20 min at room temperature, the mixture was centrifuged at 12,000 rpm (4 °C, 5 min). The supernatant was collected, and the extraction procedure was repeated. Combined supernatants were concentrated under N_2_ stream and reconstituted in 100 μL dichloromethane. The final extract was filtered through 0.22 μm syringe filters and stored in amber vials for UPLC-MS/MS analysis.

Samples were separated using a YMC C_30_ column (3 μm, 100 × 2.0 mm) maintained at 28 °C. The mobile phase consisted of (A) methanol/acetonitrile (25:75, *v*/*v*) with 0.1% formic acid and 0.01% BHT, and (B) methyl tert-butyl ether with 0.01% BHT. The gradient program is shown in Table S1. The flow rate was 0.8 mL/min with a 2 μL injection volume. The mass spectrometric detection employed an Atmospheric Pressure Chemical Ionization (APCI) source (350 °C) with 25 psi curtain gas. On the Q-Trap 6500+ system (AB SCIEX, Framingham, MA, USA), each ion pair was detected with optimized declustering potential and collision energy. Quantification was performed using multiple reaction monitoring (MRM) mode with external calibration curves.

### 2.6. Extraction and Digestion of Proteins

Frozen samples (−80 °C) of YSK, SSK, YSC, and SSC were transferred to grinding tubes while maintaining cryogenic state. An appropriate volume of protein lysis buffer (8 M urea + 1% sodium dodecyl sulfate) supplemented with protease inhibitors was added. Samples were homogenized using a cryogenic grinder with three cycles of 40 s each, followed by on-ice lysis for 30 min with intermittent vortex mixing. After lysis, the mixture was centrifuged at 16,000× *g* (4 °C, 30 min), and the supernatant was collected for protein concentration determination using BCA Protein Assay Kit (Thermo Scientific, Waltham, MA, USA). A solution containing 100 μg protein was re-suspended with triethylammonium bicarbonate buffer (TEAB) and tris (2-carboxyethyl) phosphine (TCEP), followed by incubation at 37 °C for 60 min. Subsequently, 40 mM iodoacetamide (IAA) was added for alkylation reaction at room temperature in darkness. Pre-chilled acetone (acetone/sample, 6:1, *v*/*v*) was added to each tube for protein precipitation at −20 °C. After centrifugation at 10,000× *g* for 20 min, the pellet was collected and redissolved in 100 μL of 100 mM TEAB. Trypsin was added at a 1:50 (enzyme/protein, *w*/*w*) ratio for overnight digestion at 37 °C. The peptides obtained by digestion were lyophilized using a vacuum concentrator (JX-ZLN-B, Shanghai Jingxin Industrial Development Co., Ltd., Shanghai, China). The dried peptides were reconstituted in 0.1% trifluoroacetic acid (TFA) and desalted using Hydrophilic-Lipophilic Balance Solid Phase Extraction (HLB SPE) columns (SOLAµ^TM^SPE, Thermo Scientific, Waltham, MA, USA). After vacuum concentration, peptide quantification was performed using a NANO DROP ONE (Thermo Scientific) kit.

### 2.7. LC-MS/MS Detection of Peptides

Peptides were dissolved in MS loading buffer at equal amounts, spiked with 10 × iRT peptides (Biognosys, Schlieren, Switzerland), and analyzed by LC-MS/MS using a Q Exactive™ HF-X mass spectrometer coupled to an EASY-nLC 1200 system (Thermo Scientific, Waltham, MA, USA). All analytical procedures were performed at Majorbio Bio-Pharm Technology Co., Ltd. (Shanghai, China). Briefly, peptides were separated on an EASY-nLC 1200 system using a C18 column (75 μm × 25 cm) with mobile phase A (2% acetonitrile/0.1% formic acid) and B (80% acetonitrile/0.1% formic acid). The total run time of chromatography was set at 120 min. MS analysis was performed on a Q-Exactive HF-X in data-independent acquisition (DIA) mode with an *m*/*z* range of 300–1500 and higher-energy collisional dissociation (HCD) fragmentation.

### 2.8. Protein Identification and Bioinformatic Analysis

The DIA raw data were analyzed in Spectronaut software (version 19) [39]. The reference database was downloaded from the *Larimichthys crocea* UniProt proteome (https://www.uniprot.org/taxonomy/215358 (accessed on 20 June 2024)). The quantification parameters set in the analysis are detailed in Table S2. Bioinformatic analysis of proteomic data was performed using the Majorbio Cloud platform (https://cloud.majorbio.com). *p* value and fold change (FC) of proteins between groups were calculated using R package (version 4.1.1) “*t*-test”. Proteins meeting the criteria of *p* < 0.05 and FC > 1.2 or FC < 0.83 were identified as differential abundance proteins (DAPs). Gene Ontology (GO) annotation analysis (http://geneontology.org/) was conducted for all identified proteins, and Kyoto Encyclopedia of Genes and Genomes (KEGG) pathway analysis (http://www.genome.jp/kegg/ (accessed on 18 July 2025)) was employed to investigate metabolic pathways associated with the DAPs.

### 2.9. Statistical Analysis

Quantitative data were presented as means ± standard deviation (SD). Prior to parametric tests, the assumptions were verified. The normality of data distribution was assessed using the Shapiro–Wilk test, and the homogeneity of variances was evaluated using Levene’s test. The one-way analysis of variance (ANOVA) method (performed in IBM SPSS Statistics 22 software, version 22) was used for significant differences analysis, with *p* < 0.05 or *p* < 0.01 or *p* < 0.001 considered statistically significant. The relationships between color difference values, the size and number of pigment cells, and total carotenoid content in large yellow croakers were performed using Pearson correlation analysis (performed in OriginPro 2021 software, version 9.8.0.200). The bivariate normal distribution assumption for Pearson correlation was confirmed by visual inspection of scatter plots and normality tests of residuals.

## 3. Results

### 3.1. Body Color Varies Across Different Body Regions

The color difference measurement results (Table 1) were analyzed by one-way ANOVA. A significant main effect of skin region was found for L* value (F(2, 27) = 208.94, *p* < 0.001). Post-hoc comparisons revealed that ventral skins exhibited the highest lightness (L*), which was significantly greater than that of all other regions (all *p* < 0.001), while dorsal skins showed the darkest coloration (significantly lower than ventral and caudal skins, both *p* < 0.001). Similarly, for a* values (F(2, 27) = 27.59, *p* < 0.001), the ventral skins displayed significantly higher redness than both the dorsal and caudal regions (both *p* < 0.001), with no significant difference observed between the latter two (*p* = 0.08). Regarding yellowness (b*), a significant regional difference was also observed (F(2, 27) = 531.79, *p* < 0.001). Post-hoc tests indicated that the ventral skins had the highest value, which was significantly greater than that of dorsal skins (*p* < 0.001) and caudal skins (*p* < 0.001), whereas the dorsal skins demonstrated the least yellowness (significantly lower than both ventral and caudal skins, both *p* < 0.001).

### 3.2. Observation of Chromatophore Types in Large Yellow Croaker

**Figure 2:**
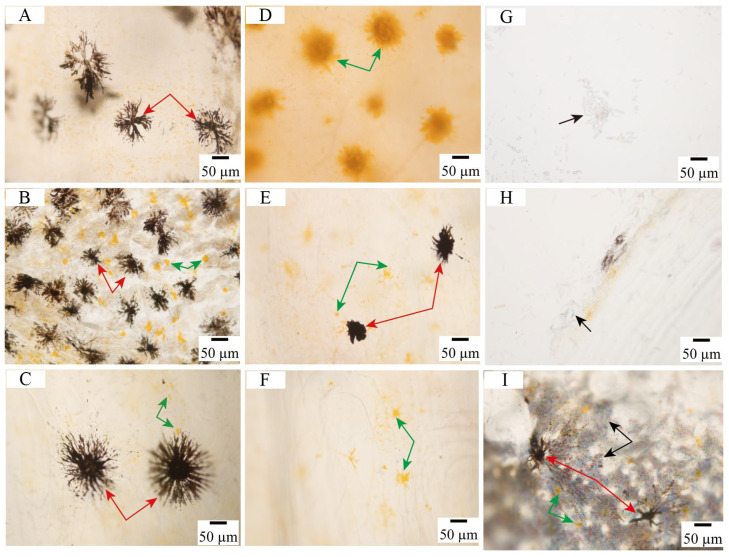
Chromatophores observed in the skins and scales of large yellow croakers. (**A**,**B**): dorsal skins; (**C**,**E**,**F**): ventral fin rays; (**D**): ventral skin; (**G**): caudal scales; (**H**,**I**): dorsal scales. Red arrows indicate melanophores, green arrows indicate xanthophores, and black arrows indicate iridophores.

Three chromatophore types, namely melanophores, xanthophores, and iridophores, were identified in the large yellow croaker. Melanophores and xanthophores were distributed in skins, scales, and fins, with melanophores generally exhibiting larger cell size than other chromatophores and coloration ranging from brown to black. Two distinct melanophore morphologies were observed: Type I featured small cell bodies with few short dendritic branches (Figure 2E); Type II displayed numerous slender dendritic branches extending radially (Figure 2A,C). Xanthophores in the ventral skin appeared as densely pigmented clustered aggregates (Figure 2D), whereas those in both the dorsal skin and the ventral fin rays showed faint pigmentation with small cell size (Figure 2B,F). Iridophores, primarily localized on scales (Figure 2G–I), formed compact arrays composed of hexagonal guanine platelets. These platelets were closely packed and variably oriented, creating the observed reflective surfaces. Comparison of different chromatophore types in large yellow croaker was shown in Table S3.

### 3.3. Distribution of Chromatophores in the Skin, Scales, and Fins of Large Yellow Croaker

**Figure 3:**
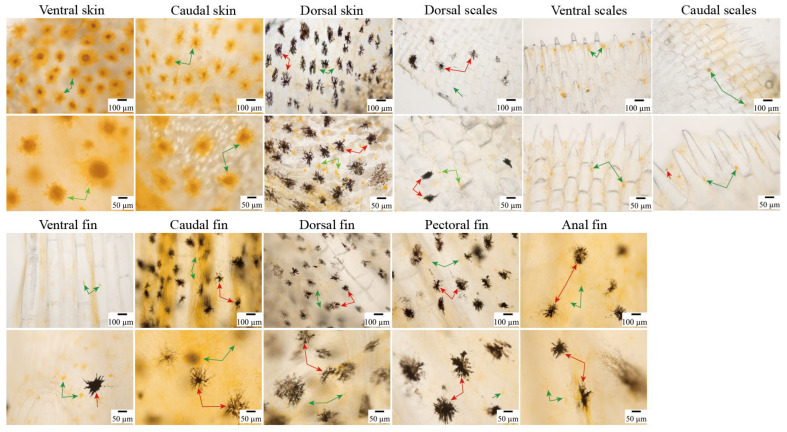
Distribution of chromatophores in the skin, scales, and fins of large yellow croakers. Red arrows indicate melanophores, and green arrows indicate xanthophores.

Xanthophores were observed in the ventral, dorsal, and caudal skins of large yellow croakers (Figure 3). The ventral and caudal skins appeared to have a higher density of these cells compared to the dorsal skins, with xanthophores densely clustered and lump-like in morphology, appearing darker in color. The xanthophores in the ventral skin appeared to be the largest, whereas those in the dorsal skin exhibited a sparse distribution, appearing as tiny, punctate light-yellow granules. No melanophores were observed in either the ventral skin or caudal skin (Figure 3). The dorsal skin harbored abundant melanophores of three morphological types: exhibiting prominent dendrites with sparse but robust branching; demonstrating more extensive dendritic branches; appearing in clustered form with nearly absent dendritic branches. Xanthophores were observed in the scales of dorsal, ventral, and caudal regions in large yellow croakers. Dorsal scales contained very few xanthophores, while those in ventral and caudal scales were smaller in size but more dispersed compared to their dermal counterparts, appearing as punctate spots. Melanophores were distributed at a lower density on ventral and caudal scales compared to dorsal scales, which maintained a high density (Figure 3).

Both xanthophores and melanophores were observed in all fins (Figure 3). The xanthophores appeared to be smaller in size compared to melanophores. In the anal and caudal fins, xanthophores appeared as fine granules uniformly dispersed with deeper pigmentation, whereas in the dorsal, pectoral, and ventral fins, they exhibited non-uniform granular distribution with lighter coloration. Melanophores were sparsely distributed in the ventral fins.

### 3.4. Diurnal Color Variation and Xanthophore Dynamics in Large Yellow Croaker

**Figure 4:**
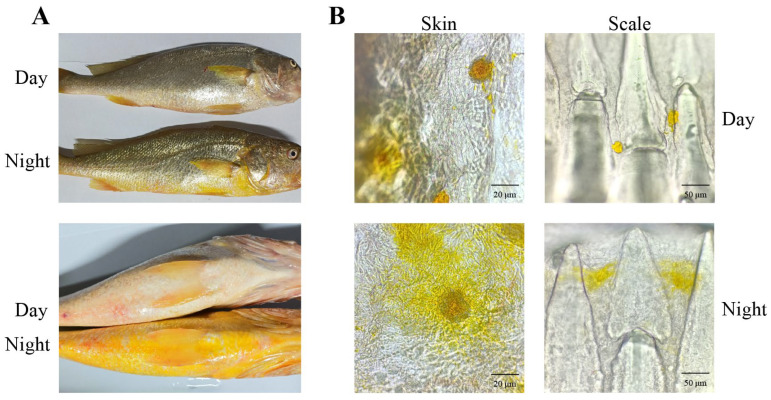
Diurnal changes in body coloration and xanthophores of the large yellow croaker. (**A**), diurnal change of the body surface color; Scale bar = 1:3.03; (**B**), diurnal dynamics of xanthophore morphology in the skin and scale tissues.

The large yellow croaker displays silvery-white coloration on both lateral surfaces and ventral regions during the daytime, and demonstrates golden-yellow at night-time, with the ventral color contrast appearing markedly stronger at night than during the daytime (Figure 4A). Results indicated a distinct nocturnal change in the morphology of xanthophores in the ventral skin and scales (Figure 4B). In daylight, their pigment granules (xanthosomes) contracted to into aggregated clusters, whereas at night, the xanthosomes dispersed extensively, with increased dendritic branching. This expansion of pigment-filled cells resulted in a prominent golden-yellow body coloration.

### 3.5. Carotenoids Content in Large Yellow Croaker

#### 3.5.1. Total Carotenoid Content in Various Tissues of Large Yellow Croaker

**Figure 5:**
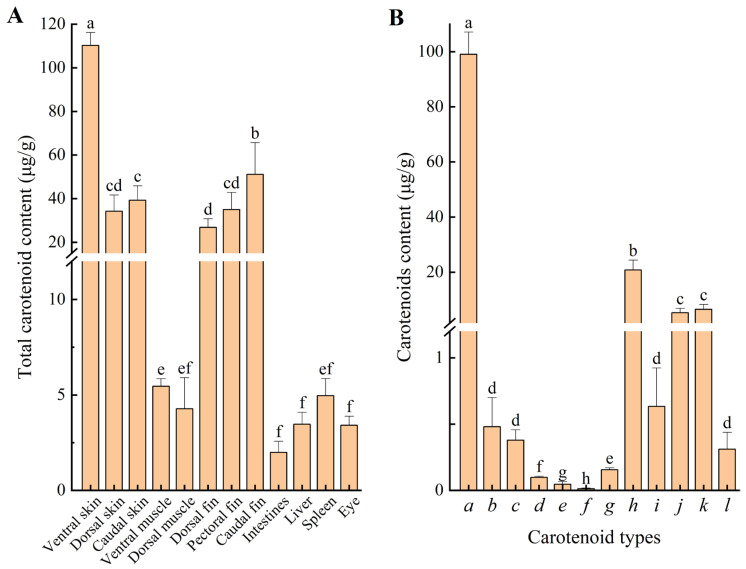
Carotenoids content in large yellow croakers. (**A**), total carotenoid content in various tissues of fish; (**B**), composition and content of carotenoids in ventral skin of fish. Different lowercase letters on the column indicated significant differences (*p* < 0.05). *a*–*l*, represent the names of each detected carotenoid: *a*, 5,6-epoxy-lutein-caprate-palmitate; *b*, Rubixanthin palmitate; *c*, Violaxanthin myristate; *d*, Antheraxanthin dipalmitate; *e*, 5,6-epoxy-lutein dilaurate; *f*, Violaxanthin laurate; *g*, Zeaxanthin myristoleate; *h*, Zeaxanthin palmitate; *i*, β-cryptoxanthin palmitate; *j*, Lutein dioleate; *k*, Lycopene; *l*, Phytofluene.

Carotenoids were distributed across various tissues, but they were primarily deposited in the skin, followed by the fins (Figure 5A). The ventral skin contained the highest total carotenoid content (110.25 μg/g), with the caudal fin having the second highest content. The contents in the dorsal skin, caudal skin, dorsal fin, and pectoral fin showed no significant differences (*p* > 0.05), but were significantly higher than those found in the muscles and other visceral tissues (*p* < 0.05, Figure 5A).

#### 3.5.2. Composition and Content of Carotenoids in Large Yellow Croaker

Based on the finding that the ventral skin of large yellow croaker contained the highest total carotenoid content, we analyzed the composition and content of carotenoids in ventral skin using UPLC-MS/MS. Figure 5B revealed the presence of 12 carotenoids in the ventral skin of large yellow croakers. Among these, 10 were xanthophylls; representative compounds included 5,6-epoxy-lutein-caprate-palmitate, rubixanthin palmitate, and violaxanthin myristate. Additionally, two carotenes were identified: lycopene and phytofluene. The xanthophyll content was 126.91 μg/g, accounting for 94.91% of the total carotenoids, representing the primary pigment source in large yellow croakers. The carotene content was 6.80 μg/g, comprising 5.09% of the total carotenoids. The predominant xanthophylls in the ventral skin of large yellow croakers was 5,6-epoxy-lutein-caprate-palmitate (99.04 μg/g), constituting 74.07% of total carotenoids, followed by zeaxanthin palmitate (20.77 μg/g, 15.53%). Lycopene and phytofluene were present at content of 6.49 μg/g (4.86%) and 0.31 μg/g (0.23%), respectively.

### 3.6. Relationships Among Body Color, Xanthophore, and Carotenoid Content in Large Yellow Croaker

**Figure 6:**
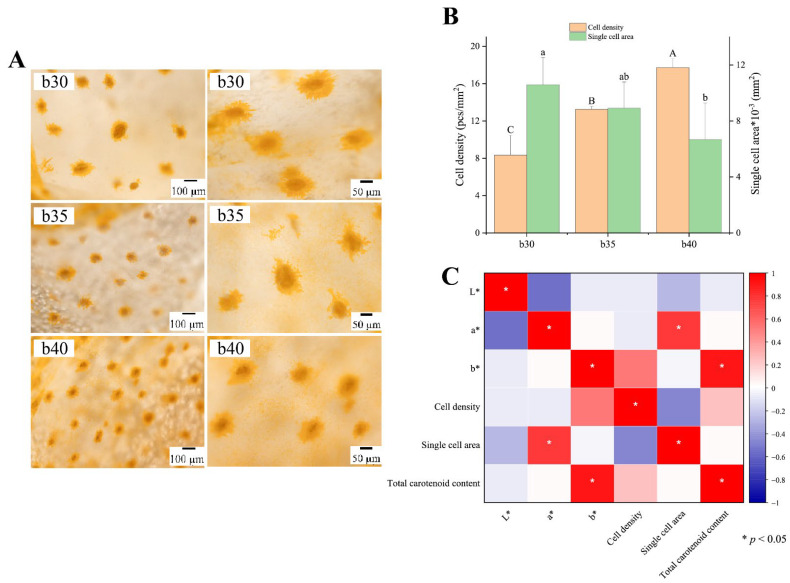
The relationship between body color and xanthophore, carotenoid content in large yellow croakers (*n* = 3). (**A**), xanthophores were observed in fish exhibiting varying b* values; (**B**), comparison of cell density and single cell area of xanthophores in fish exhibiting varying b* values; (**C**), correlation analysis between color difference values, cell density and area of xanthophores, and total carotenoid content in large yellow croakers. b30, b35, and b40 represent mean b* values of 30, 35, and 40, respectively. Different letters on the column indicated significant differences (*p* < 0.05); * *p* < 0.05 means the correlation between two variables has reached a statistically significant level.

To analyze the impact of xanthophores on the yellowness coloration of large yellow croaker, we first compared differences in xanthophore density and cellular area among specimens exhibiting varying b* values (Figure 6A). Sampling sites of ventral skin with mean b* values of 30, 35, and 40 were selected and their corresponding xanthophore density and individual cell area were quantified. Figure 6B demonstrated that higher b* values correlated with increased xanthophore density, and fish in the b40 group exhibited significantly higher number of xanthophores than b35 and b30 groups (*p* < 0.05). In contrast to the result of cell density, the single cell area showed an inverse pattern, with the b40 group exhibiting significantly smaller cellular areas than the b30 group (*p* < 0.05, Figure 6B).

Based on the results from Figure 6B, we hypothesized a potential correlation between b* values, xanthophore density and area, subsequently performing a correlation analysis as presented in Figure 6C. As shown in Figure 6C, b* values had a significantly positive correlation with total carotenoid content (*R* = 0.91, *p* < 0.05), but only a positive, non-significant trend with xanthophores cell density (*R* = 0.53, *p* > 0.05). In addition, single cell area was positively correlated with a* values (*R* = 0.77, *p* < 0.05). The results indicated that xanthophores and pigment granules showed significant correlation with large yellow croaker body coloration.

### 3.7. Comparative Analysis of Identified Protein Samples

To further elucidate the mechanisms underlying diurnal variations in the xanthophores of the large yellow croaker, we conducted a comparative proteomic analysis of ventral scales and skin tissues collected during day and night cycles. As shown in Figure S1A, 5266 proteins in YSK, 5243 proteins in SSK, 5164 proteins in YSC, and 5180 proteins in SSC were identified, of which 5070 proteins were common to all four groups. The number of proteins common to both YSK and SSK was 5235, while 5113 proteins were common to both YSC and SSC samples. Correlation analysis of protein expression (Figure S1B) demonstrated high reproducibility within biological replicates (all intra-group correlations > 0.90). While correlations between skin groups (YSK versus SSK) and between scale groups (YSC versus SSC) were above 0.67, those between skin and scale groups were all below 0.50. This pattern of inter-group variation was corroborated by PCA (Figure S1C), which showed a clear separation between skin and scale samples. Both analyses consistently indicate that sample type (skin versus scale) is the primary driver of proteomic variation in this study.

### 3.8. Differential Abundance Proteins (DAPs) Analysis

**Figure 7:**
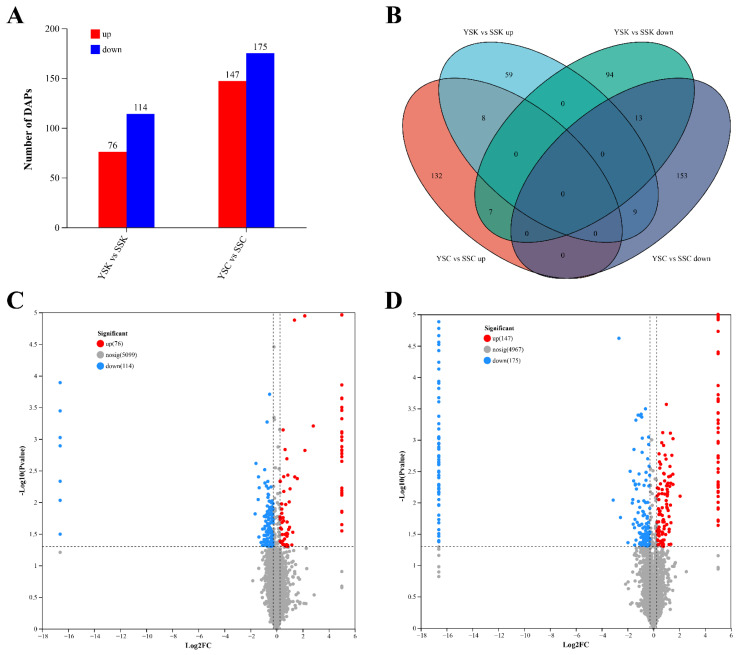
Statistical analysis of DAPs among groups. (**A**), statistics chart of DAPs; (**B**), Venn diagram of DAPs; (**C**), volcano map of DAPs between YSK and SSK; (**D**), volcano map of DAPs between YSC and SSC. Up: up-regulated DAPs; down: down-regulated DAPs. Sample groups: YSK, the ventral skin tissues of fish caught at night; SSK, the ventral skin tissues of fish caught in the daytime; YSC, the scale tissues of fish caught at night; SSC, the scale tissues of fish caught in the daytime.

Screening for DAPs was performed using FC > 1.2 or FC < 0.83 and a significance level of 0.05. The numbers of DAPs in the skin (YSK versus SSK) and scales (YSC versus SSC) of large yellow croakers were 190 and 322, respectively (Figure 7A). Compared to SSK samples, 76 proteins were up-regulated and 114 proteins were down-regulated in YSK samples. Compared to SSC samples, 147 proteins were up-regulated and 175 proteins were down-regulated in YSC samples. Among these, eight proteins were up-regulated and 13 proteins were down-regulated in both YSK and YSC samples (Figure 7B). The volcano maps of DAPs in YSK versus SSK and YSC versus SSC groups are shown in Figure 7C,D. Detailed information of partial DAPs are listed in Table S4.

### 3.9. Gene Ontology (GO) Annotations Analysis of DAPs

**Figure 8:**
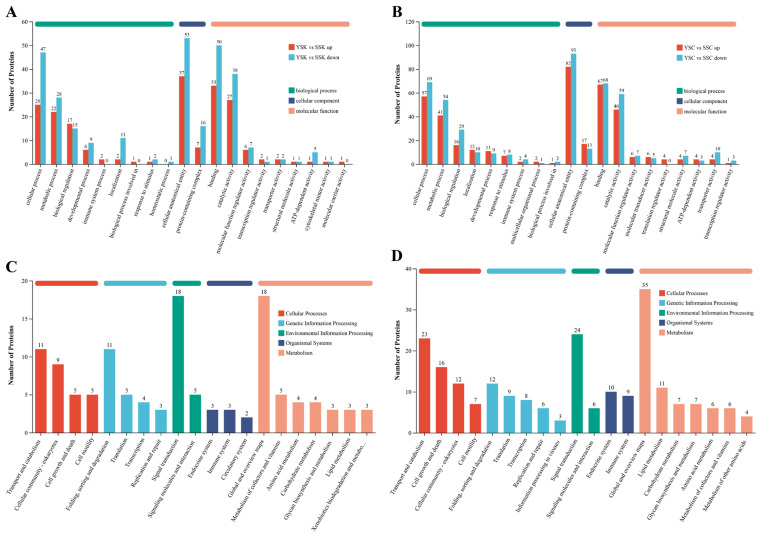
GO annotations (Level 2) and KEGG pathways analysis of DAPs in the skin and scales of large yellow croakers. (**A**), GO annotations of DAPs in the YSK versus SSK group; (**B**), GO annotations of DAPs in the YSC versus SSC group; (**C**), KEGG pathways analysis of DAPs in the YSK versus SSK group; (**D**), KEGG pathways analysis of DAPs in the YSC versus SSC group.

The DAPs were searched against the GO database to perform GO functional analysis. The level 2 annotation results of these DAPs in the GO database are shown in Figure 8A,B. GO functional annotations can be grouped into three broad categories: biological process (BP), cellular component (CC), and molecular function (MF). Figure 8 shows that DAPs in the ventral skin and scales of large yellow croakers during diurnal cycles were involved in similar GO functions. Both up- and down-regulated DAPs were primarily involved in cellular process, metabolic process, biological regulation, location, cellular anatomical entity, protein-containing complex, binding, and catalytic activity. GO enrichment analysis (Figure S2A) revealed several significantly enriched GO terms, such as signaling receptor activity, molecular transducer activity, transmembrane signaling receptor activity, and membrane (YSC versus SSC). In the YSK versus SSK group, representative enriched GO terms were embryonic organ development, cell migration, cell motility, and transmembrane receptor protein serine/threonine kinase signaling pathway (Figure S2B).

### 3.10. KEGG Pathways Analysis of DAPs

**Figure 9:**
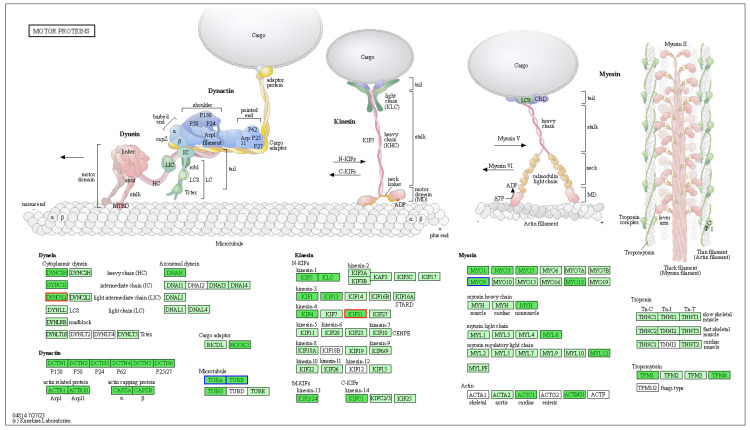
KEGG pathway annotation of motor proteins in the YSC versus SSC group. Red border indicates up-regulated proteins and blue border indicates down-regulated proteins. The figure was collected from the public database (https://www.kegg.jp/pathway/lco04814 (accessed on 23 July 2025)).

The top 20 KEGG pathways of DAPs in the skin and scales of large yellow croakers are shown in Figure 8C,D. In the YSK versus SSK group, DAPs were mainly involved in KEGG pathways of transport and catabolism, cell motility, signal transduction, signaling molecules and interaction, endocrine system, metabolism of cofactors and vitamins, carbohydrate metabolism and lipid metabolism (Figure 8C). DAPs identified in the scale group (YSC versus SSC, Figure 8D) were enriched in identical KEGG pathways to those in the skin group (YSK versus SSK), but exhibited quantitative differences in the number of associated DAPs per pathway between these tissue types. Among these pathways, the calcium signaling pathway, regulation of actin cytoskeleton, and motor proteins (Table S5) were reported to be associated with pigment granule movement [34]. The KEGG pathway map of motor proteins (Figure 9) revealed five significantly altered proteins in YSC group compared with SSC group: two up-regulated—kinesin family member 21 (KIF21) and dynein 1 light intermediate chain 1 (DYNC1L1), and three down-regulated—*α*-tubulin (TUBA), *β*-tubulin (TUBB), and myosin-IX (MYO9).

## 4. Discussion

The body coloration of fish is formed by chromatophores, influenced by the type, number, distribution, and pigment content within these cells [40], and is also regulated by neural, hormonal, and environmental factors [41]. Even within the same fish species, the types, density, and arrangement patterns of chromatophores differ across various tissues and parts such as the skin, scales, and fin rays. In recent years, observations of fish chromatophores have primarily focused on the skin and scales. For instance, the chromatophores of zebrafish and turbot include three distinct types: xanthophores, melanophores, and iridophores [7,8], while crucian carp skin possesses four: melanophores, xanthophores, erythrophores, and iridophores [9]. The skin of the mandarinfish (*Siniperca chuatsi*) contains melanophores, xanthophores, and iridophores, while its scales possess melanophores and xanthophores. These chromatophores are unevenly distributed across different body parts and vary in size, brightness, and spacing, contributing to the diverse body coloration [42]. In recent years, microscopic investigations into the body coloration of the large yellow croaker have gradually increased. Microscopic observation of the color of large yellow croakers revealed the presence of four types of chromatophores: melanophores, xanthophores, erythrophores, and iridophores. The distribution, morphology, and quantity of each cell type differed, and their pigment granule content also varied [43,44]. Han et al. [10] identified the presence of xanthophores and melanophores in the dorsal skin of the large yellow croaker. Shi et al. [11] further confirmed the existence of iridophores in addition to xanthophores and melanophores through microstructural examination of both dorsal and ventral regions. Chromatophores observed in our research aligned with those reported previously [11], with both detecting xanthophores, melanophores, and iridophores. In our study, erythrophores were not observed in the tissues of large yellow croaker. The distribution patterns of the other three chromatophores types were consistent with those observations [43]: xanthophores were primarily located in the ventral skin, melanophores were mainly found in the dorsal skin and scales, and iridophores were predominantly located in scales.

Microscopic observations revealed that the number and morphology of chromatophores significantly influence the body coloration of the large yellow croaker. The dorsal region exhibited a higher density of melanophores, with their black pigment granules arranged in a radiate pattern, resulting in a predominantly light-black dorsal coloration. In contrast, the ventral region displayed densely distributed xanthophores with dispersed pigment granules. Additionally, no melanophores were observed in the ventral skin or scales. The morphology of melanophores in all fins resembled that observed in the skin of large yellow croakers. As aquatic organisms, fish possess a countershading coloration pattern—darker dorsally and lighter ventrally—which aids in evading predation from above-water predators [45]. The observed differences in chromatophores composition between the dorsal and ventral regions in the large yellow croaker laid the foundation for this countershading adaptation. Studies have shown that the interactions between chromatophores influence the final presentation of fish body coloration. It was found that the formation of zebrafish stripes initiated with the iridophores in the compartments of the stripes [46]. Subsequently, iridophores facilitated the formation of melanophore stripes and recruited xanthophores to aggregate. Xanthophores, in turn, repelled melanophores. This dynamic ultimately gave rise to the characteristic alternating dark and light stripe patterns on the zebrafish body surface [46]. We speculate that the differential distribution of chromatophores along the dorsal-ventral axis in large yellow croaker may result from interactions among xanthophores, melanophores, and iridophores.

The chromatophores in fish exhibit diverse coloration due to the presence of distinct pigmentary substances, which typically include melanin, carotenoids, pteridines, and guanine crystals [5]. The yellow/red coloration in fish results from carotenoids and pteridines contained within specialized chromatophores: xanthophores (yellow pigment cells) and erythrophores (red pigment cells) [47]. Carotenoids in fish are primarily deposited in the skin, muscle, scales, fins, eyes, liver, and gonads [48]. Total carotenoid content varies significantly across tissues. In *Lutjanus erythropterus*, the highest concentrations occur in the skin and eyes [49]. Salmonids such as *Salmo salar* and *Oncorhynchus mykiss* exhibit elevated carotenoid levels in muscle tissue. Juvenile *Salvelinus alpinus* deposit carotenoids predominantly in muscle, followed sequentially by skin, liver, and gonads [50]. In this study, the skin and fins were identified as the major deposition sites of total carotenoids in large yellow croakers, with the highest content detected specifically in the ventral skin and the caudal fin. This distribution pattern aligns closely with the observed phenotypic coloration: regions such as the ventral skin and caudal fin exhibit more intense yellow pigmentation, which is driven by both the high local density of xanthophores and the localized accumulation of carotenoids within them [12]. These findings are consistent with our results of color difference analysis performed earlier (Table 1), confirming that spatial variation in pigment concentration directly influences visible color attributes.

Carotenoids are the primary pigments responsible for the yellow to red coloration in most fish [23]. Although fish cannot synthesize carotenoids, they can modify the ingested carotenoids through metabolic processes, converting them into different carotenoid forms [51]. Different fish species vary in their metabolic capabilities for carotenoids, resulting in variations in the specific types of carotenoids that play the dominant functional role within their bodies [23]. In this study, 12 carotenoids were identified in the ventral skin of large yellow croakers, predominantly in esterified form. Lutein esters constituted the most abundant fraction, existing as 5,6-epoxy-lutein-caprate-palmitate (74.07% of total carotenoids) and lutein dioleate. The findings aligned with those reported by Zheng et al. [38], who similarly identified the two lutein esters as the predominant carotenoids in the skin of large yellow croaker. Lutein esters and zeaxanthin esters are known for imparting a typical yellow pigmentation, which can give channel catfish yellow coloration [52]. In this study, lycopene accounted for 4.86% of the total carotenoid content in large yellow croaker skin and contributed to its red chromatic components. Yi et al. [53] found that dietary supplementation of lutein significantly enhanced skin yellowness and carotenoid deposition in the large yellow croaker, while elevated dietary canthaxanthin levels increased skin redness. Additionally, their study demonstrated a significant linear correlation between dermal carotenoid content and b* values in large yellow croaker—consistent with our results of a strong positive correlation (*R* = 0.91, *p* < 0.05) between skin b* value and total carotenoid concentration. Both dietary xanthophyll and astaxanthin could significantly enhance skin pigmentation and carotenoids content in lager yellow croaker [54]. Elucidating the composition and concentration of carotenoids in large yellow croaker skin can provide direct scientific basis for implementing dietary interventions—specifically through aquafeed supplementation with targeted carotenoid types and ratios—to optimize or maintain desirable yellow pigmentation in fish.

Studies confirmed that golden-yellow coloration of large yellow croakers originated from carotenoids within xanthophores [54]. We conducted a comparative analysis of diurnal variations in xanthophores distribution across ventral skin and ventral scales of the large yellow croaker. We found that the fish exhibited a diurnal color shift from silvery-white during the day to golden-yellow at night. This color change resulted from the nocturnal dispersion and daytime aggregation of xanthosomes within the ventral xanthophores. Under dark conditions, localized light irradiation caused the yellow coloration of large yellow croakers to change to silvery white [34]. Under white light conditions, partial shading treatment induced a shift from silvery white to yellow in the shaded areas [34]. In vitro experiments with cultured xanthophores demonstrated rapid pigment aggregation within 2 min of light exposure [55]. Following shading treatment, the pigment granules underwent dispersion, achieving complete diffusion in approximately 45 min [55]. Based on the findings of our study, it is further confirmed that the skin coloration of large yellow croaker is influenced by light exposure. The contraction and transport of xanthosomes within xanthophores are responsible for the diurnal color variation observed in this species.

Proteomic analysis revealed that in the YSC versus SSC group, representative up-regulated DAPs included 39S ribosomal protein L16 (RP-L16, A0A6G0IZP7), A0A6G0J836, dynein light intermediate chain (DYNC1LI, A0A6G0HSB0), serine/threonine-protein kinase (A0A6G0JA29), and KIF21 (A0A6G0HRD6). Similarly, key down-regulated DAPs included tubulin alpha chain (TUBA, A0A6G0IMT5), secretory carrier-associated membrane protein (MYO9, A0A6G0J1B2), A0A6G0HUI8, A0A0F8AEG2, and tubulin beta chain (TUBB, A0A6G0II50). RP-L16 and A0A6G0J836 are ribosomal proteins, which are involved in translation, ribosomal structure and biogenesis [56]. DYNC1LI and KIF21 (kinesin-like protein) are motor proteins that can directionally transport intracellular cargos, such as protein complexes and membranous organelles [57,58]. MYO9, a class IX myosin motor protein, regulates actin cytoskeleton dynamics and undergoes directed movement along actin filaments [59]. In the YSK versus SSK group, there were also two down-regulated DAPs belonging to myosin, namely MYL6 (H9E9C5) and A0A6G0HUI8. MYL6 (myosin light chain 6), an essential light chain binding myosin heavy chain and smooth muscle myosin, stabilizes the myosin structure [60]. Low-density lipoprotein receptor (LDLR, A0A6G0J8K5) was up-regulated in the YSK group compared to SSK and contributes to carotenoid homeostasis and transport [61].

Proteomics results showed that DAPs altered in the ventral skin and scales of large yellow croakers during diurnal cycles shared common Gene Ontology (GO) functional foundations, (e.g., cellular processes, metabolism, regulation), indicating that these tissues shared core regulatory mechanisms in responding to circadian rhythms. GO enrichment analysis revealed distinct functional emphases between tissues, which may reflect their different roles. In scales, significant enrichment in signaling receptor activity (e.g., transmembrane receptors) is consistent with a potential role in environmental signal perception. We therefore speculate that scales could be a key site where light or endogenous rhythmic signals are sensed via membrane receptors (such as opsins [62,63]), potentially initiating color change responses. In contrast, the enrichment for cell migration, motility, and kinase pathways in skin aligns more closely with the regulation of effector cell behavior. This supports a plausible hypothesis that skin tissue is directly involved in executing color change through modulating chromatophore activities, which may include changes in morphology, distribution, migration, pigment granule transport in melanophores [10] and xanthophores [34,64]. The combined action of these proposed mechanisms (environmental signal perception, intracellular pathway activation, and chromatophore response) collectively drives the adaptive diurnal color changes observed in the ventral region. The results help to elucidate the regulatory mechanisms of diurnal body color variation in large yellow croaker at the proteomic level.

Studies have demonstrated that treating xanthophores of large yellow croakers with depolymerizing agents of microfilaments and microtubules disrupted light-induced translocation of pigment granules within these cells, indicating that xanthophore granule transport relies on both actin filaments and microtubules [64]. Motor proteins, which utilize energy from ATP hydrolysis to propel themselves directionally along microtubules or actin filaments, mediate intracellular cargo transport [65]. These include microtubule-associated motors (kinesins and dyneins) and actin-based motors (myosins). Each motor protein contains a conserved domain that binds cytoskeletal tracks, and specialized cargo-binding domains that recognize macromolecular complexes [66]. Dynein inhibitor treatment impaired pigment granule aggregation in xanthophores, further confirming motor proteins’ essential role in pigment translocation [34]. Given the heterogeneous cellular composition of fish skin compared to the pigment cell-dominated architecture of scales, our analysis focused specifically on DAPs related to motor molecules in scales. Five DAPs were found significantly altered in the KEGG pathway map of motor proteins (Figure 9). KIF21, a member of the N-KIF family, transports intracellular cargo and drives movement toward the plus end of microtubules, which is directed toward the cell periphery [58]. In this study, the KIF21 expression in large yellow croaker scales was significantly higher at night than during the day. This suggests that KIF21 may participate in the transport (dispersion) of pigment granules (xanthosomes) within xanthophores towards the cell membrane at night, thereby contributing to the yellow body coloration of the fish. Dynein is a massive protein complex composed of heavy chains, intermediate chains, light intermediate chains, and light chains [57]. As a component of the dynein, DYNC1LI significantly influences the biological processes in which dynein participates. Dynein primarily moves along microtubules in a minus-end-directed manner, that is, towards the cell center [67]. Mammalian MYO9b has been demonstrated to be a unique motor protein capable of moving toward both the minus- and plus-end of actin filaments [59]. In this study, DYNC1LI expression in fish scales was higher at night than during the day. MYO9 exhibited significantly higher protein expression during the day compared to night. These findings suggest that DYNC1LI and MYO9 may not be directly involved in xanthosomes transport, but rather in maintaining their pigment distribution state. Specifically, DYNC1L1 may regulate xanthosomes distribution near the membrane, while MYO9 may govern their distribution near the cell center. Alpha-tubulin (TUBA) and beta-tubulin (TUBB) are essential proteins that constitute microtubules. These two tubulin isoforms share similar three-dimensional structures and bind tightly to form heterodimers, which serve as the fundamental subunits for microtubule assembly [68]. Microtubules have been demonstrated to participate in the transport of pigment granules within xanthophores [34,64]. They provide “tracks” for motor proteins, enabling these motors to move along the microtubules and thereby facilitate pigment transport [69]. The expression levels of TUBA and TUBB in scale tissues also exhibited significant diurnal variations, which revealed that the diurnal changes in body coloration of the large yellow croaker were not only associated with the transport activity of motor proteins, but also involved the assembly and disassembly of microtubules.

## 5. Conclusions

This study characterizes the body coloration of large yellow croaker, revealing that ventral skin-specific xanthophore abundance and lutein-dominated carotenoid deposition are pivotal for golden color formation. The aggregation and dispersion of xanthosomes within xanthophores is thought to be responsible for the light-dependent diurnal body color variation in the fish. The process of pigment granule movement may be related to specific motor proteins, such as KIF21 and DYNC1L1, and involves concomitant tubulin depolymerization and polymerization, indicating that microtubules actively participate in regulating pigment movement. The present study has inferred the role of dynamic pigment granule transport and motor proteins in the diurnal body color changes of large yellow croaker. The functional roles of the proteins discussed in this study are inferred primarily from their diurnal expression patterns and existing literature. In the future, direct experimental validation—such as cellular localization, overexpression, or knockdown of the specific proteins—is required to confirm these proposed functions.

## Figures and Tables

**Table 1:** Comparation of color difference values among different body regions in large yellow croaker.

Body Regions	L*	a*	b*
Ventral area	73.45 ± 3.21 ^a^	2.21 ± 2.63 ^a^	37.01 ± 2.21 ^a^
Dorsal area	48.04 ± 2.12 ^c^	−3.23 ± 0.36 ^b^	7.89 ± 1.92 ^c^
Caudal peduncle	53.25 ± 3.32 ^b^	−1.83 ± 1.27 ^b^	19.27 ± 1.88 ^b^

Note: Data are presented as mean ± SD (*n* = 10). L* stands for lightness; a* stands for redness to greenness; b* stands for yellowness to blueness. Means with different superscript letters within the same column are significantly different (*p* < 0.001).

## Data Availability

Data will be made available on request.

## Supplementary Materials

The following supporting information can be downloaded at: https://www.mdpi.com/article/10.3390/ani16030353/s1, Table S1. Gradient elution program for HPLC. Table S2. Protein Database Search Parameters. Table S3. Comparison of different chromatophore types in large yellow croaker. Table S4. Partial differential abundance proteins (DAPs) in YSC vs SSC samples or YSK vs SSK samples. Table S5. KEGG pathways (YSC vs SSC group) of Cell motility, including Motor proteins and Regulation of actin cytoskeleton. Figure S1. Comparative analysis of identified protein samples in large yellow croakers (*n* = 3). Figure S2. GO enrichment analysis (Level 2) of DAPs in the scales (A, YSC vs SSC) and skin (B, YSK vs SSK) of large yellow croakers.
