# Supplementary material for: Body Coloration Characterization and Proteomic Analysis of Diurnal Color Variation in Farmed Larimichthys crocea
Source: Animals (Basel). 2026 Jan 23;16(3):353. doi: 10.3390/ani16030353 (PMC12896436; doi:10.3390/ani16030353)
Supplement: Supplementary file 1 [file animals-16-00353-s001.zip › animals-4097766-supplementary.pdf]

Supplementary materials

Table S1 Gradient elution program for HPLC.

Run time (min)	Mobile phase A (%)	Mobile phase B (%)
0	100	0
3	100	0
5	30	70
9	5	95
10	100	0
11	100	0

Note: Mobile phase A, methanol/acetonitrile (25:75, v/v) with 0.1% formic acid and 0.01% BHT; Mobile phase B, methyl tert-butyl ether with 0.01% BHT.

Table S2 Protein Database Search Parameters.

Parameters	Set
Peptide length range	7-52
Enzyme	trypsin/P
maximum missed cleavage site	2
Fixed modification	Carbamidomethyl (C)
Variable modifications	Oxidation (M); Acetylation (Protein N-term)
Protin FDR	≤ 0.01
Peptide FDR	≤ 0.01
Peptide Confidence	≥ 99%
XIC width	≤ 75 ppm

Table S3 Comparison of different chromatophore types in large yellow croaker

Chromatophore types	Cell morphology	Distribution
Melanophores	Exhibiting larger cell size; Coloration ranging from brown to black; Type I: small cell bodies with few short dendritic branches; Type II: numerous slender dendritic branches extending radially	Skins of dorsal; Scales of dorsal and caudal; All fins
Xanthophores	Densely pigmented clustered aggregates or faint pigmentation with small cell size	Skins of ventral, dorsal, and caudal; Scales of dorsal, ventral, and caudal; All fins
Iridophores	Formed compact arrays composed of hexagonal guanine platelets and these platelets were closely packed and variably oriented	Scales

Table S4 Partial differential abundance proteins (DAPs) in YSC vs SSC samples or YSK vs SSK samples.

Accession	Description	Fold change	Fold change	Regulate	KO Name
		YSC vs SSC	YSK vs SSK		
A0A6G0IZP7	Large ribosomal subunit protein uL16m	1.96	-	up	RP-L16
A0A6G0J836	Translation initiation factor IF-2	32.00	-	up	infB
A0A6G0HSB0	Dynein light intermediate chain	32.00	-	up	DYNC1LI
A0A6G0JA29	Serine/threonine-protein kinase PLK	32.00	-	up	PLK1
A0A6G0HRD6	Kinesin-like protein KIF21A	32.00	-	up	KIF21
A0A6G0IMT5	Tubulin alpha chain	0.79	-	down	TUBA
A0A6G0J1B2	Secretory carrier-associated membrane protein	0.57	-	down	MYO9
A0A6G0HUI8	Myosin-11	0.3647	-	down	-
A0A0F8AEG2	Small ribosomal subunit protein mS25	1.00E-05	-	down	MRPS25
A0A6G0II50	Tubulin beta chain	1.00E-05	-	down	TUBB
H9E9C5	Myosin light polypeptide 6	-	0.79	down	MYL6
A0A6G0HUI8	Myosin-11	-	0.61	down	-
A0A6G0J8K5	Low-density lipoprotein receptor	-	32.00	up	LDLR

Table S5 KEGG pathways (YSC vs SSC group) of Cell motility, including Motor proteins and Regulation of actin cytoskeleton.

Second Category	Pathway ID	Description	All up proteins [a]	All down proteins [b]
Cell motility	lco04814	Motor proteins	A0A6G0HSB0; A0A6G0HRD6	A0A6G0II50; A0A6G0J1B2; A0A6G0IMT5
Cell motility	lco04810	Regulation of actin cytoskeleton	-	A0A6G0HWE7; A0A6G0I8A6

Note: [a] A list of up-regulated DAPs annotated to this pathway.

[b] A list of down-regulated DAPs annotated to this pathway.

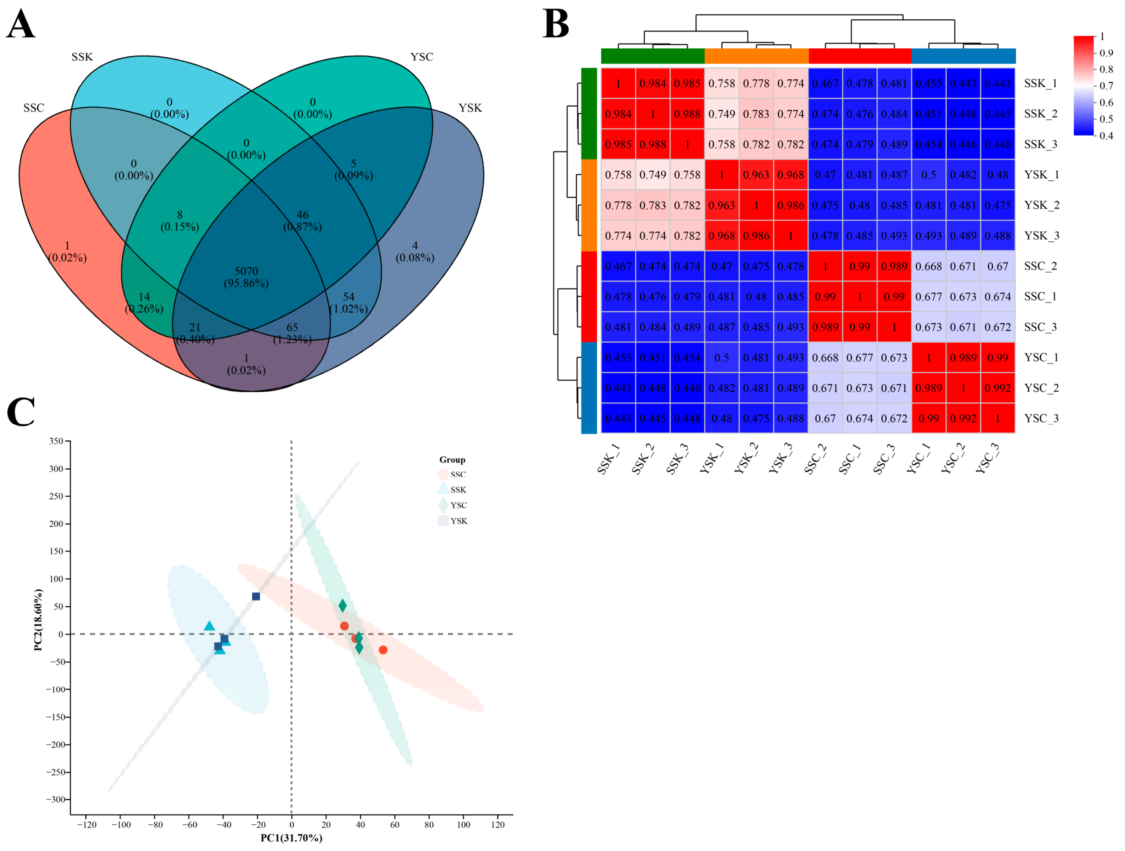

Figure S1. Comparative analysis of identified protein samples in large yellow croakers (n = 3). A, venn diagram of identified proteins in different samples; B, correlation heatmap of proteins expression in different samples; C, PCA analysis of samples. Sample groups: YSK, the ventral skin tissues of fish caught at night; SSK, the ventral skin tissues of fish caught in the daytime; YSC, the scale tissues of fish caught at night; SSC, the scale tissues of fish caught in the daytime.

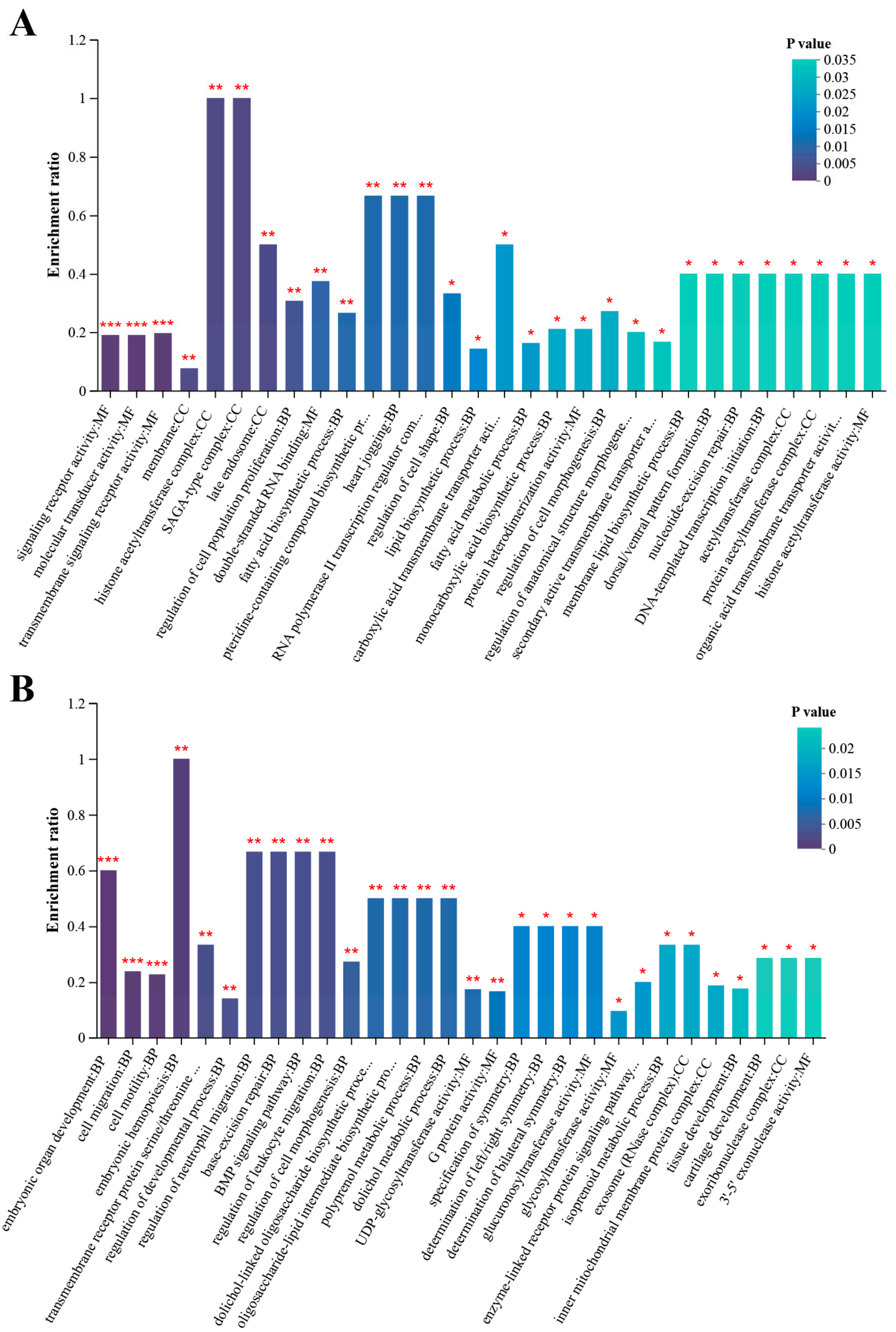

Figure S2. GO enrichment analysis (Level 2) of DAPs in the scales (A, YSC vs SSC) and skin (B, YSK vs SSK) of large yellow croakers. The color gradient of the columns represents the significance of enrichment, with $P < 0.05$ denoted by *, $P < 0.01$ denoted by **, and $P < 0.001$ denoted by ***.
